# Leveraging advances in machine learning for the robust classification and interpretation of networks

**DOI:** 10.1098/rsos.240458

**Published:** 2025-04-30

**Authors:** Raima Carol Appaw, Nicholas M. Fountain-Jones, Michael A. Charleston

**Affiliations:** ^1^ Department of Mathematics, University of Tasmania College of Sciences and Engineering, Sandy Bay, Tasmania, Australia; ^2^ School of Natural Sciences, University of Tasmania, Hobart, Tasmania, Australia

**Keywords:** network science, classification, machine learning, generative graph models

## Abstract

The ability to simulate realistic networks based on empirical data is an important task across scientific disciplines, from epidemiology to computer science. Often, simulation approaches involve selecting a suitable network generative model such as Erdös–Rényi or small-world. However, few tools are available to quantify if a particular generative model is suitable for capturing a given network structure or organization. We utilize advances in interpretable machine learning to classify simulated networks by our generative models based on various network attributes, using both primary features and their interactions. Our study underscores the significance of specific network features and their interactions in distinguishing generative models, comprehending complex network structures and the formation of real-world networks.

## 1. Introduction

Real-world network data derived from physical systems such as ecological food webs, biochemical pathways, genetic interactions, animal social behaviour and biological processes capture complex relationships and address fundamental questions about species adaptability, ecosystem dynamics, pathogen dynamics, social dynamics and genetic regulatory networks [1–6]. The multidimensional nature and dynamic interactions among variables over time in these systems pose a challenge to their classification. Traditional classification methods (such as decision trees, support vector machines, k-nearest neighbour and logistic regression) struggle to capture these complexities effectively [7–10]. Moreover, the lack of interpretability in machine learning models further compounds this challenge.

Machine learning techniques, including supervised learning, have been applied to the classification of real-world networks into theoretical network models, also known as generative models. Jansen *et al*. [11] introduced the Alternating Decision Tree (ADT), an unsupervised algorithm designed for network classification by focusing on model selection through synthetic network fitting to real network data. Their approach involved generating 1000 graphs and extracting various network features, training the ADT using nine classifiers for effective model selection. Similarly, Ikehara *et al.* [12] conducted a study on 986 real networks and 575 generated networks, utilizing binary classification with graph features. Barnett *et al.* [13] used a random forest algorithm for network classification, manually selecting individual graph features, while Canning *et al.* [14] proposed a random forest approach for classifying 529 networks. Although these efforts to address network similarity, feature identification and optimization, the studies did not consider the impact of feature interaction on classification. Graph similarity metrics, such as degree distribution and clustering, have been utilized to compare graph centrality measures between real-world and theoretical networks [9,10,15–17]. Although some measures show effectiveness in classifying real-world networks, there remains potential for enhancing the efficiency of these methods.

Various network models such as Erdös–Rényi (ER), scale-free (SF), small-world (SW), stochastic block model (SBM) and spatial networks have been used to analyse real-world data [1,18–23]. These network models are straightforward to simulate and facilitate the testing of new and diverse network-related hypotheses. Using these network models characterized by different network structures and dynamics has set the stage for pioneering research, offering the potential to classify empirical networks effectively [2,3,24–27]. An important inquiry in our study revolves around the suitability of a spatial network model that reproduces structural characteristics observed in real-world networks. Despite their potential for estimating vertex similarity from graph structures, making them superior to other models for classification purposes [11], spatial models are often underutilized in predictive modelling frameworks. In a spatial model, individuals are positioned in space, where edge formation depends on the distance between individuals [1,11]. Spatial representation within networks enables the quantification of social space by identifying communities or individuals with similar characteristics [11]. While scientists utilize network models to classify real-world networks, the challenge lies in identifying the most effective network model for the classification task. Various methods have been employed to tackle this issue; however, none have systematically considered the interactions between network features in the classification of empirical networks [9,10,15–17,24].

To this end, we use a wide range of theoretical network models, each representing different structural properties observed in real-world networks. We simulated networks from five different generative models, spanning a wide range of sizes and parameter combinations. This approach ensured that the simulated networks exhibited key target properties such as sparsity, SW characteristics, clustering coefficient (transitivity), mean path length and power-law degree distribution [1,21,28]. As a result, our study has two main contributions:

(i) We identify new and specific features that can predict the different generative models, including spectral measures—like the (normalized) Fiedler value and spectral radius, in prediction of ER and SW networks, respectively.
(ii) We established thresholds for feature values where interactions among features become important for network prediction.

To achieve these goals, we employed recent model interpretation techniques like Shapley additive explanations (SHAP) [29,30] and Friedman and Popescu’s H-statistics [31]. We hope that our modern approach to investigating these features and interactions for classification will benefit future researchers.

**Figure 1.**
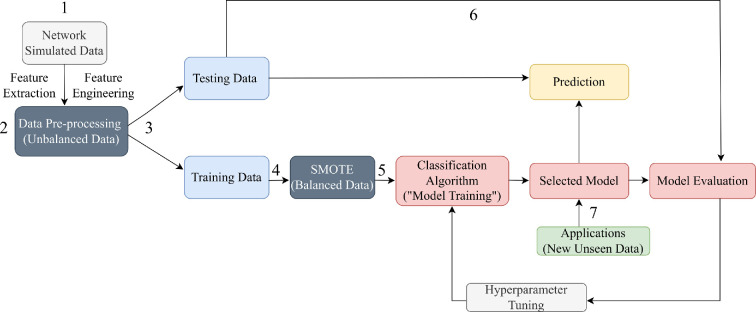
Overview of the network classification method. (1) Many examples of networks are simulated with the generative models (light grey) and their features are calculated and important features retained during data preprocessing (dark grey). (2) Data pre-processing includes feature extraction and engineering. (3) Data are split into test and training datasets (blue). (4) SMOTE with the tidymodel framework in R software [32] is applied to correct for class imbalance. (5) Models are trained, and the best classification model is selected (pink), once the models’ hyperparameters have been tuned (light grey). (6) The test data are used for model prediction (yellow) and evaluation (pink). (7) The final selected model is applied to new datasets (green) to predict the generative model class (yellow).

Overall, our approach (see figure 1) aims to enhance our understanding of the generative models shaping network formation, revealing critical features and their interaction influencing the observed structures in diverse physical systems. We also applied our model to real-world networks [19,33,34] demonstrating the practical utility of our approach.

### 1.1. Notation and description of network features

We introduce the notation used in the description of network features used for classification and provide descriptions below. A graph is defined as a set of *nodes* (referred to as individuals or actors in contact networks) and *edges* that represent connections between pairs of nodes. Mathematically, a *graph* G=(V,E) consists of a finite set V of nodes and a set E of pairs of nodes ($v_{i},v_{j}$), known as edges.

Two nodes $v_{1}$ and $v_{2}$ are called *adjacent* if there is an edge connecting them. The *degree* of a vertex v, denoted deg(v), is the number of edges connecting that vertex to other vertices. In our studies, we focus mainly on undirected graphs, where edges have no direction—that is, edge ($v_{i},v_{j}$) is equivalent to ($v_{j},v_{i}$) for all i,j. The graph G can be mathematically represented using various types of matrices such as the *adjacency matrix* and the *Laplacian matrix*. The adjacency matrix of a graph G with n nodes is represented by an n×n symmetric matrix A(G) and is defined as


$$A=[a_{ij}]:=\left\{ \begin{array}{cl} 1 & \text{if }v_{i},v_{j}\text{ are adjacent;}\\ 0 & \text{otherwise.} \end{array}\right.$$

The Laplacian matrix is the difference between the degree matrix and the adjacency matrix, given as L=D−A. Additionally, we denote the normalized Laplacian matrix as $L=D^{-\frac{1}{2}}LD^{-\frac{1}{2}}$, where D and $D^{-\frac{1}{2}}$ are defined below.

The degree matrix D(G) is an n×n diagonal matrix of graph G defined as


$$D=[d_{ij}]:=\left\{ \begin{array}{cl} deg(v_{i}) & \text{if }i=j\text{;}\\ 0 & \text{otherwise.} \end{array}\right.$$

A closely related matrix of D is $D^{\frac{-1}{2}}$


$$D^{\frac{-1}{2}}=[d_{ij}]:=\left\{ \begin{array}{cl} \frac{1}{\sqrt{deg(v_{i})}} & \text{if }i=j\text{;}\\ 0 & \text{otherwise.} \end{array}\right.$$

An important object in graph analysis is the *spectrum* of each of the various matrices associated with G. This is an ordered list of the *eigenvalues* of the matrix M in question, written $\lambda_{1}\leq \lambda_{2}\leq \lambda_{3}\leq \cdots \leq \lambda_{n}$.

The *j*th eigenvalue $\lambda_{j}$ of any of the above matrices encodes information associated with the *j*th eigenvector, which we denote as $\psi_{j}$. The second smallest non-trivial eigenvalue of the Laplacian and normalized Laplacian matrix are called the *Fiedler* and *normalized Fiedler* values, respectively. Similarly, the largest eigenvalue of the adjacency matrix is called the *spectral radius*. These three eigenvalues are important in capturing structural properties of complex networks and graphs and have been studied in the application of graph theory and network analysis in many fields [7,16,35].

With these foundational concepts defined, we can describe the graph features necessary for graph classification (refer to electronic supplementary material, Definition of Graph Features, for additional details).

## 2. Data generation and feature engineering

### 2.1. Theoretical networks (generative models)

We utilized the *igraph* package within the R software to generate approximately 442 142 networks, varying in size from 50 to 1000 nodes. Among these simulated networks, 309 499 were allocated for training purposes, while 132 643 were reserved for testing, using a split ratio of 70 : 30.

For each network type, we generated over 200 instances for each parameter combination. We achieved this by adjusting the configuration algorithms and/or model parameters for different network sizes, allowing us to simulate networks with various numbers of edges. Some network models had wider parameter ranges, resulting in class imbalance (some networks have more simulated data than others). To address the class imbalance, we applied the synthetic minority over-sampling technique (SMOTE) with the *tidymodel* framework in R software [12,32,36]. This involves randomly reconstructing new network instances for all minority classes to equalize the data points, aligning them with the largest class [12,32]. After applying SMOTE, the training data totalled 674 885. Overall, we had 807 528 networks across the five generative models used for classification purposes.

For a breakdown of parameter ranges for the generative models, see electronic supplementary material, table S1. The generative models are described as follows.

#### 2.1.1. Erdös–Rényi random graph

We synthesized random graphs using the ER graph model [18]. In ER graphs, each node has a fixed probability $P^{\left(ER\right)}$ of connecting with every other node in the graph [18]. For our random graphs, we assigned the connection probability $P^{\left(ER\right)}$ drawn from a uniform distribution: $P^{\left(ER\right)}∼U(0.1,0.9)$. In ER networks, the spatial position of individuals is irrelevant with connections forming at uniform random [28]. Therefore, these networks tend to be homogeneous and characterized by low clustering coefficient (transitivity) and short path lengths [28]. The degree distribution of an ER random graph follows a Poisson distribution [28].

#### 2.1.2. Small world

We generated SW networks using the Watts–Strogatz SW model [19] within the *igraph* package. This involved rewiring edges of a regular lattice graph, with rewiring probabilities $P^{\left(SW\right)}$ set at 0.1 and 0.3. The parameter $l^{s}$, representing the neighbourhood within the lattice, ranged from 1 to 35. In SW networks, each edge has a probability $P^{\left(SW\right)}$ of being rewired to different random nodes. Higher values of $P^{\left(SW\right)}$ influence the graph’s structure while maintaining the total number of edges. The SW network is characterized by the short path length of the ER networks and the high clustering of the lattice network [28]. The degree distribution of the SW network is similar to the ER network [28].

#### 2.1.3. Spatial graph

We constructed a spatial model using a threshold distance parameter, denoted as r, in the range of 0.1≤r≤0.9. We randomly distributed N points within a unit square, and pairs of points were connected if their Euclidean distance was within the threshold distance r. As r increased, the number of connections in this network also increased. By altering the spatial arrangement of individuals in a spatial network, a diverse array of networks can be created, spanning from densely clustered lattices to SW configurations to fully interconnected random networks [28]. The degree distribution of spatial networks follows a Poisson distribution [28]. Spatial networks are characterized by a high degree of heterogeneity [28].

#### 2.1.4. Scale-free

We constructed SF networks using the Barabasi–Albert preferential attachment model [20], where nodes are added one at a time. In this model, each new node $v_{i}$ attaches up to m pre-existing nodes $v_{j}$ according to the following probability formula: $P^{\left(BA\right)}$ = $P(v_{j})$= $deg(v_{j}{)}^{\alpha }$ / $\sum_{i}deg(v_{i}{)}^{\alpha }$. The existing nodes to which the new node is attached are selected randomly, with replacement, using these probabilities, potentially resulting in fewer than m. Our simulations considered values of m∈{1,2,3,4,5,....,35} and the preferential attachment parameter α∈{1,2,3}. In network analysis, individuals with high connectivity, known as superspreaders, play a crucial role in propagation or diffusion processes like disease transmission [28]. Including superspreaders in networks is essential for capturing the complexities of propagation processes. SF networks offer a solution by enabling extreme levels of heterogeneity [28]. Similar to the random networks, in SF networks, the spatial position of individuals is ignored in forming connections or links [28]. Their degree distribution follows a power-law pattern [20,28].

#### 2.1.5. Stochastic block model

SBMs extend random graph models by introducing explicit community structures [22–24]. In SBMs, nodes are partitioned into communities or ‘blocks’, where nodes have stronger connections within the same community than between different communities [22]. The parameters of SBMs include the number of nodes N, the number of disjoint blocks s, each with a size $C_{j}$ for j=1,…,s, into which the nodes are partitioned and an s×s symmetric edge probability matrix P. This matrix defines the within- and between-community connection probabilities for the nodes. For our experiments, we generated graphs under this model with s=2 blocks, $C_{1}=0.4N$, $C_{2}=N-C_{1}$ and varying P. Specifically, the connection probability within block i was drawn from a uniform probability density function U(0.5,0.9), and the probability P between blocks was drawn from U(0.1,0.4).

### 2.2. Empirical networks (model application)

The empirical network data used for model application were obtained from published works [19,33,34]. These networks consist of the electrical power grid of the western United States [19], the badger social network [33], macromolecular networks such as protein and metabolic networks [37,38], biological gene networks and the distribution of the sub-webs within ecological food webs [34,39,40].

### 2.3. Feature selection

We computed graph features for both theoretical and empirical networks and used these features as feature vectors in our classification tasks. These features capture various aspects of network structure. For a detailed definition of the graph features, refer to electronic supplementary material, Additional Graph Definitions. In total, we manually selected 18 features, excluding features such as maximum degree, number of triangles, maximum triangle and total number of triangles, which were correlated with the number of nodes (order). The 18 selected features are degree centrality, eigen centrality, closeness centrality, betweenness centrality, degree assortativity coefficient, Fiedler value, normalized Fiedler value, spectral radius, modularity, clustering coefficient (transitivity), mean degree, diameter, mean eccentricity, mean path length, graph energy, minimum cut, order (number of nodes) and number of edges. The feature set we selected is a subset of the features utilized by [41]; however, their feature set does not include the spectral radius or normalized Fiedler. Similarly, [24] did not incorporate either the (normalized) Fiedler value or the spectral radius in their analysis.

**Figure 2.**
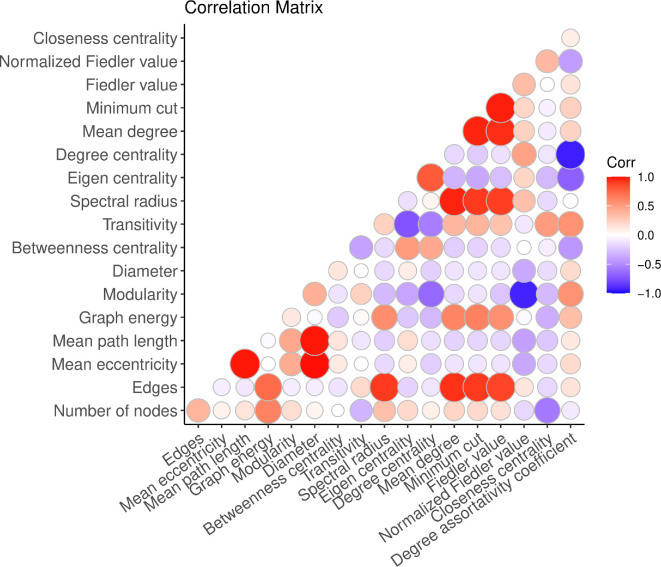
Correlation among selected network features. Most features are correlated (using the Pearson correlation). The strength and direction of correlation are indicated by size and colour. For example, Fiedler value and mean degree are highly positively correlated, and normalized Fiedler has a higher negative correlation with modularity.

We investigated the interdependence among the selected features using a correlation matrix to detect potential multicollinearity [42,43]. Spearman’s correlation coefficient (ρ) [43,44] was utilized to identify the features with high correlations (figure 2).

To address feature correlation and select relevant features for our classification tasks, we employed the ‘Boruta’ feature selection algorithm [45]. Boruta identifies all relevant features by iteratively comparing the importance of each feature with that of a ‘shadow’ feature created by permuting the original feature values [45]. This process helps distinguish truly important features from those that are not [45]. Despite significant correlations among the features shown in figure 2, the Boruta algorithm retained all 18 graph features. This suggests that each feature, as well as the collective set, is important for predicting the generative models. Additionally, SHAP values provide further insights by highlighting which features contribute most to the model’s final predictions. This combined approach ensures the retention of all relevant features, avoiding the loss of potentially critical information due to feature correlation and enhancing our ability to interpret the importance of individual features in the model’s final outcome.

## 3. Model description and interpretation tools

We used the *random forest* (RF) and *boosted tree* (XGBM) classification algorithms for training and testing, utilizing features extracted from various network instances and parameters, spanning the generative models. Both RF and XGBM are tree-based algorithms; XGBM fits trees sequentially to correct errors from the previous one, while trees in the RF model are fitted in parallel independently with each iteration [5]. Feature engineering was applied to the extracted features to determine which features to retain or eliminate, enhancing algorithm efficiency, performance and overall predictive model accuracy [5]. The training and testing sets were divided into a 70 : 30 ratio, with both testing and training procedures leveraging the *tidymodel* framework within the R environment [32]. Tunable hyperparameters included ‘mtry’ and ‘trees’ for RF, and ‘mtry’, ‘learning rate’, ‘trees’, and ‘tree depth’ for XGBM. In the *tidymodel* framework, various combinations of hyperparameter values can easily be built using packages such as *tune*, *dials* and *tune_race_anova* [32,46,47]. We employed the *grid_maximum_entropy* method from the *tune_race_anova* package to tune hyperparameters in the *tidymodel* framework, generating a grid search with points selected to maximize entropy for both XGBM and RF models. In total, 400 and 160 000 combinations of hyperparameter values were generated for RF and XGBM, respectively.

In addition, a repeated k-fold cross-validation (with k set to 10), randomly splitting datasets into k groups, was utilized in the training process to estimate model performance and evaluate the impact of tuning hyperparameters, such as learning rate, on RF and XGBM model performance [5]. This approach aids in selecting the optimal model by considering factors like sensitivity and specificity via the confusion matrix for classification models [5]. Cross-validation prevents overfitting and artificial inflation of accuracy by assessing model performance on different data subsets [5].

### 3.1. Shapley additive explanations model-agnostic interpretation method

SHAP, based on game theory principles, assigns distinct importance weights to individual features according to their contributions to model output and their interactions with other features [8,29,30]. Computation of SHAP values involves solving a system of linear equations using a specialized weighted linear regression approximation method, quantifying how much importance the model attributes to each feature in its predictions [8,29,30]. SHAP analysis provides both local and global feature importance and interpretations, including waterfall plots, variable importance and summary plots, SHAP interaction plots and dependency plots. In context, global feature importance assesses each feature’s overall contribution to a model’s performance or predictions across the entire dataset, while local feature importance evaluates an individual feature’s contribution to a single data instance. SHAP model output is typically expressed in log-odds units [48]. Overall, SHAP values can provide important insights into a model's performance in highlighting the most impactful features for each predicted class category [29].

The local and global SHAP techniques employed in our study to investigate the main feature effects are described below:

(i) The SHAP waterfall plot serves as a local feature importance visualization, displaying the contribution of each feature to a model’s output for a specific data instance. This plot helps visualize how individual features influence a particular prediction, aiding in understanding the model’s decision-making process. The *x*-axis represents SHAP values’ magnitude, indicating the expected predicted values of the model’s output. The *y*-axis, represented by horizontal bars, corresponds to the features and their contributions to shifting the model’s output from a reference (base) value. Bar length signifies the SHAP value’s magnitude for a specific feature, and bar colour indicates whether the feature’s effect on the prediction pushes it toward a higher (positive, red bars pointing right) or lower (negative, blue bars pointing left) model output.
(ii) The SHAP global feature importance can be presented as a feature summary plot, or global variable importance plot, or a combination of both. These plots illustrate the direction and magnitude of each feature’s impact on a model’s final predictions. They can be visualized as bars, similar to variable importance bar charts, or as dots representing feature summaries. Dots may be coloured to indicate the feature’s value (high or low), while bar length corresponds to each feature’s strength [29,30,49]. The global feature importance can also be explored with the SHAP dependency plot [29,30,49].
  The SHAP plots identify the most influential model features for individual and global predictions, offering insights into the model’s behaviour across the dataset [29,30,49]. Note that in many cases, only a small range of these values is important to classification, such as mean eccentricity for spatial networks and mean path length for the SBM; conversely, transitivity is important across a wide range of values when predicting ER. Features can exhibit negative or positive SHAP values, reflecting their impact on the model’s prediction. High positive SHAP values exert a greater positive impact on the prediction, while large negative SHAP values correspond to negative predictions. For example, a positive SHAP value for high transitivity and low mean eccentricity tends to favour spatial networks, whereas a positive SHAP value for low modularity and high normalized Fiedler suggests ER networks.
(iii) SHAP dependency plots further explore global feature importance illustrating the relationship between a feature and its impact on the model’s prediction using scatter plots [29,30,49]. These plots complement SHAP feature effect and importance plots by revealing whether a feature has a linear, monotonic or more complex relationship with the model’s prediction. Together, they offer detailed insights into how individual feature values influence the model’s final output or prediction [29,30,49].
(iv) The SHAP two-dimensional (2D) dependency plots are used to explore feature interactions and visualize the combined effects of two features on a model’s predictions [30]. These plots help in identifying and understanding the shape of the joint effects, revealing complex relationships between features. In these plots, point colours can represent different features or the predicted output, providing additional layers of insight [30,49]. This visualization technique is valuable for diagnosing and interpreting the interplay between features, making it easier to see how they jointly influence the model’s behaviour.

#### 3.1.1. H-statistics

Friedman and Popescu’s H-statistics, detailed in [31], serve as vital tools for evaluating the performance of machine learning algorithms in classification or regression tasks. These statistics identify significant variables or features involved in interactions, quantifying the strength and degree of these interactions and capturing the key variables influencing a model’s predictions. In addition, we also calculated a global statistic denoted as *H*${,}^{2}$, introduced in the same GitHub resource at https://github.com/ModelOriented/hstats#background. In summary, these measures provide a valuable technique for quantifying and gaining insights into the complex landscape of feature interactions within our classification model. We describe these measures below:

(i) *H*${,}^{2}$: Describes the proportion of prediction variability not explained by main effects [50].
(ii) *H*${,}_{i}^{2}$: Describes the proportion of the variability in prediction explained by interactions with feature i (overall interaction effect for individual feature) [31].
(iii) *H*${,}_{ij}^{2}$: Describes the proportion of the variability in prediction explained by the pairwise interaction effect between features i and j [31].
(iv) *H*${,}_{ijk}^{2}$: Describes the proportion of the variability in prediction explained by the three-way interaction effect among features i, j and k [31].

## 4. Results

Our results demonstrate that both RF and XGBM achieved a nearly 100% accuracy rate and an AUC (area under the ROC curve) score of near one across the k-fold cross-validation resamples. In addition, both specificity and sensitivity were close to 100%, showcasing the high classification performance of our model. Further analysis focused on the XGBM due to its superior speed, scalability and robustness when handling large datasets and real-time prediction tasks compared with RF.

### 4.1. Main and interaction effects

For all generative models, a substantial proportion of prediction variability remains unexplained by the sum of main effects $H^{2}$ alone (table 1). We proceed to describe the main network features influencing the prediction of the generative models, and the strength, degree and form of their interactions with other features in predicting the generative models.

**Table 1.** Performance of our classification pipeline when making predictions with test data. These metrics provide insights into how well the model is performing in terms of correctly identifying true positives, true negatives, false positives and false negatives. They are calculated by comparing the model’s predictions with the actual ground-truth labels in the test dataset. This table also shows the proportion of the prediction variability unexplained by the main effect *H*^2^ for ER: Erdös–Rényi; SBM: stochastic block model; SF: scale-free; Sp: spatial; SW: small-world.

truth								
		ER	SBM	SF	Sp	SW	recall	*H*^*2*^ (%)
predicted	ER	7421	5	0	0	13	0.9975	67
	SBM	0	4511	0	0	1	0.9997	80
	SF	1	0	57 523	0	0	0.9999	80
	Sp	0	0	0	7045	0	0.1	80
	SW	15	9	0	0	56 099	0.9995	81
	precision	0.9978	0.9969	1	1	0.9997		

#### 4.1.1. Spatial

**Figure 3.**
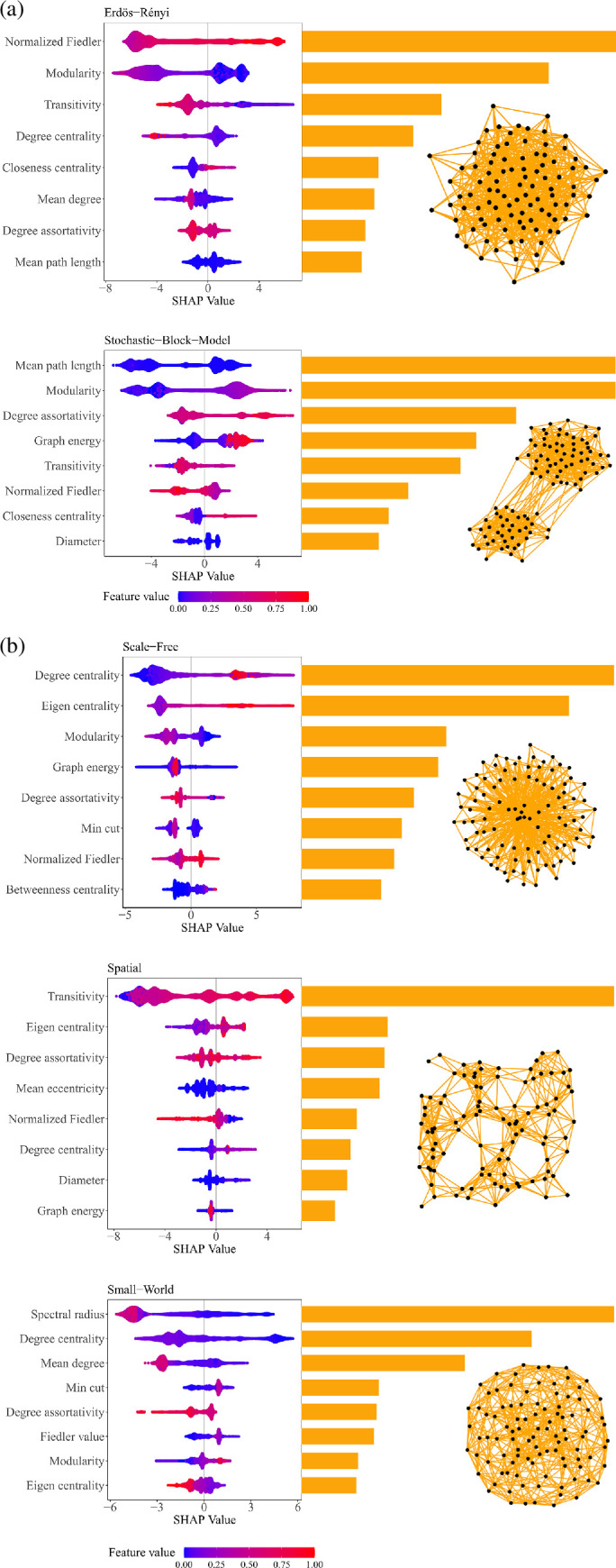
The SHAP feature importance plot provides insight into the significance of various features in predicting ER, SBM, SF, spatial and SW networks. These plots are for the entire dataset (the plots for test data only are fairly similar). Key features are positioned at the top of the plot and are represented by longer orange bars, indicating their stronger importance in influencing the model’s outcome. Notable predictors for the network types include normalized Fiedler, modularity, degree centrality, transitivity and spectral radius. In the feature effect plot, blue indicates lower feature values, while red denotes higher values, across the population of all network instances.

**Figure 4.**
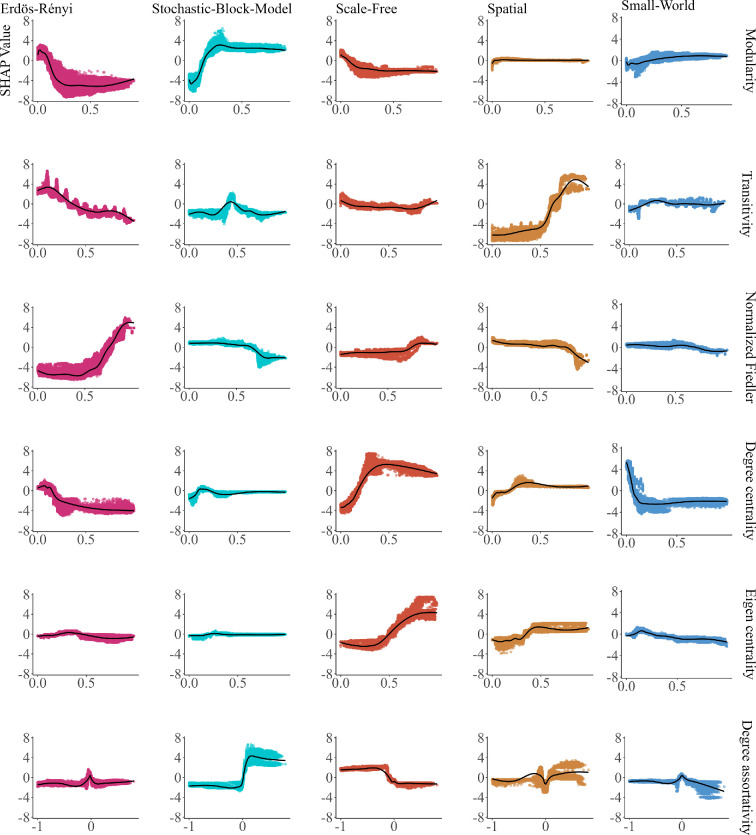
SHAP dependency plot showing the scatter plot of the relationship between each feature and its importance in predicting the final output of the ER, SBM, SF, spatial and SW generative models across different levels or values. The *y*-axis typically represents the SHAP value, which quantifies the impact of the feature on the model’s prediction, while the *x*-axis shows the feature’s value. This plot provides insights into how the model’s prediction changes as the feature’s value varies, for each class separately. The direction and magnitude of the SHAP values across the different classes aid in discerning how important the features are for each class prediction and whether its effect is consistent across all classes or varies. Overall, this plot aids in understanding the model’s behaviour and the relative importance of features across different classes, providing valuable insights into the model’s overall decision-making process for all features across all model classes.

Our investigation unveils transitivity as the strongest positive predictor for spatial networks on both global and local scales (figures 3b and 4; electronic supplementary material, figures S3–S5). Higher eigen centrality values, a positive degree assortativity coefficient and a smaller mean eccentricity and mean path length are additional features that play key roles in predicting this class (figures 3b and 4; electronic supplementary material, figures S3–S5).

**Figure 5.**
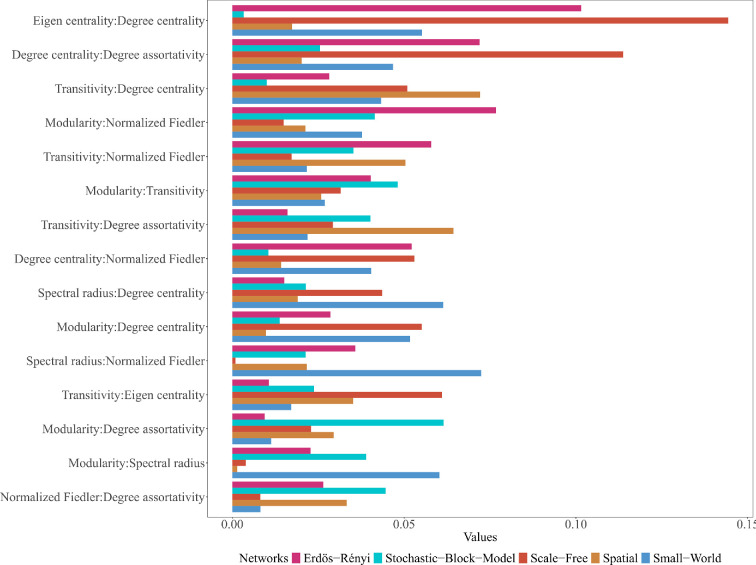
The proportion of joint effect variability of two features explained by the pairwise interactions on ER, SW, SF, spatial and SBM predictions. The *x*-axis and *y*-axis represent the feature values and pairwise feature combinations, respectively. The length of the bar associated with the pairwise features typically indicates the strength of the pairwise interaction and how the proportion of the joint effect variability explained by their pairwise interaction influences the model's final prediction across the different generative models.

Spatial networks in terms of overall feature interactions demonstrate robust interactions associated with transitivity, normalized Fiedler, degree assortativity coefficient, eigen centrality and degree centrality (electronic supplementary material, figure S1(a)). These features account for over 30% proportion of prediction variability explained by interactions on them (electronic supplementary material, figure S1(a)). Additionally, spatial networks exhibit over 6% prediction variability from pairwise interactions between transitivity and degree centrality, transitivity and degree assortativity coefficient and transitivity and eigen centrality (figure 5)

**Figure 6.**
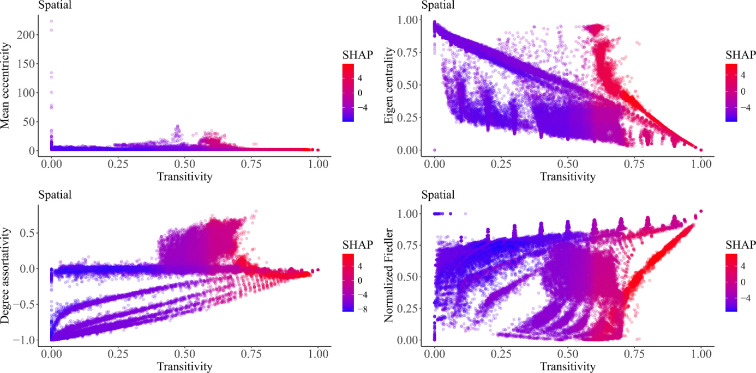
SHAP 2D dependency plot showing two-way combined effects of selected variables in predicting the spatial network. This plot shows how the predicted outcome of the spatial network depends on the values of two input features simultaneously. The plot represents the dependency between transitivity (on the *x*-axis) pairing with either mean eccentricity, or eigen centrality, or degree centrality, or degree assortativity, or modularity or spectral radius in predicting the spatial network with the other features on the *y*-axis. The colour or shading of the plot (manually set) indicates the predicted outcome or class probability for the spatial network. Overall, this plot shows at which feature values the combined effect of the two features existing simultaneously positively, or adversely affects this generative model’s class prediction. It is worth noting that the gaps in this plot signify the absence of simulated networks for specific combinations of feature values, owing to the stochastic nature of the network synthesis and the use of an unknown parameter space beyond our control.

Regarding the form of the pairwise interaction, the most significant pairwise interaction effects revolve around transitivity values of approximately 0.7 (figure 6). At this threshold, transitivity shows a strong positive pairwise interaction with other network features (figure 6). Notably, transitivity values at this threshold exhibit positive pairwise interactions with both eigen centrality values and degree centrality values below 0.5 (figure 6). A positive pairwise interaction is also observed at this 0.7 transitivity threshold alongside mean eccentricity values close to zero (figure 6). Moreover, the degree assortativity coefficient values have a strong pairwise interaction with transitivity in predicting spatial networks (figure 5)

#### 4.1.2. Small world

The spectral radius stands out as the strongest predictor of this class globally and locally (figures 3*b* and 4; electronic supplementary material, figures S3–S5). Generally, small values of the spectral radius predict this class positively, while high spectral radius does not (figures 3*b* and 4; electronic supplementary material, figures S3–S5).

On the other hand, a high degree centrality, mean degree and negative degree assortativity coefficient values predict this network negatively. However, relatively higher transitivity values and smaller values of mean path length positively predict this network (figures 3*b* and 4; electronic supplementary material, figures S3–S5).

In terms of overall interactions in the SW network, key interactions involve the spectral radius, mean path length, minimum cut, normalized Fiedler, modularity, transitivity, degree centrality, eigen centrality and degree assortativity coefficient, explaining approximately 45% of the proportion of prediction variability (electronic supplementary material, figure S1(a)). SW networks display over 6% prediction variability from pairwise interactions between spectral radius and degree centrality, spectral radius and normalized Fiedler and spectral radius and modularity (figure 5).

The form of pairwise interaction shows that when the spectral radius reaches the threshold of approximately 50 or above, it interacts with degree centrality, degree assortativity, transitivity and modularity revealing a negative pairwise relationship in predicting this class (electronic supplementary material, figure S2(c)). Furthermore, a spectral radius of approximately 50 or higher has a positive pairwise interaction with small values of mean path length in predicting the SW network (electronic supplementary material, figure S2(c)). However, we observe a different facet of the spectral radius, wherein its association with higher values of the mean degree negatively influences the SW prediction (electronic supplementary material, figure S2(c)). These pairwise interactions associated with spectral radius and other network features collectively form a complex relationship that decreases the likelihood of networks being classified as SW (electronic supplementary material, figure S2(c)). We note here that a high spectral radius interacts with the other features (i.e. degree centrality, degree assortativity, transitivity and modularity) to negatively predict this class, while a small spectral radius interacts with the features to positively predict this class (electronic supplementary material, figure S2(c)). There are other pairwise interactions between either degree centrality and other features like modularity, eigen centrality, normalized Fiedler, transitivity, degree assortativity in predicting the SW network apart from pairwise interactions between spectral radius and other features (figure 5).

While three-way interactions are weaker than pairwise interactions, they remain significant for the generative models (electronic supplementary material, figure S1(b)). Overall, investigating the main feature effects and interactions is important for predicting the generative models as it reveals various feature dependencies and interplay. These insights aid in identifying influential feature combinations, improving classification accuracy and the ability to model real-world networks more effectively.

For results on ER, SBM and SF networks, refer to Additional Results subsections of the electronic supplementary material.

### 4.2. Model application

To test the utility of our pipeline, we applied our classification model to network data of the western United States power grid [19]. Watts & Strogatz [19] determined the western United States power grid network to be a SW network, which our classification model effectively predicted. Furthermore, our classification model effectively captured the power-law (SF) degree distribution associated with macromolecular networks such as protein and metabolic networks [37,38], biological gene networks and the distribution of the sub-webs within ecological food webs [20,39,40]. In addition, our model predicted that the dynamics associated with badger social organization are spatially determined [33]. The above predictions and the data upon which they are based are available at https://github.com/araimacarol/Empirical-Network-Classifier.

Therefore, our classification pipeline demonstrates application and utility in interpreting and understanding complex structures from network data across diverse domains. We have attempted to highlight the potential of this approach for future research, for example in exploring the classification of empirical animal social networks. Moreover, we have created an interactive R-Shiny app accessible online via https://araima.shinyapps.io/Empirical-Network-Classifier. This app allows users to upload new network data and predict their generative model class using graph features employed during model training. Additionally, the app presents summaries of estimated graph features and incorporates SHAP model interpretation [29,30] and Friedman and Popescu’s H-statistics [31] for visualizing feature importance, interactions and quantifying the degree and strength of the interactions. This can aid users in understanding key features influencing the prediction of the generative model class by the model and the relevance of their interactions for the model’s prediction.

## 5. Discussion

Our machine learning approach provides a powerful method to accurately predict generative models for empirical networks and interpret model outputs. Additionally, we leveraged advances in machine learning interpretation tools like SHAP [29,30] and Friedman and Popescu’s H-statistics [31] to explore feature effects and their interactions in distinguishing between generative models. This reveals varying feature sets associated with each model and enhances the robustness of predictions for generative models.

Our findings highlight the absence of consistent patterns associated with features across models, suggesting diverse feature requirements for accurate prediction for different models. Likewise, the interaction among distinctive features, including spectral measures such as the (normalized) Fiedler value, spectral radius and eigen centrality, alongside other network properties like transitivity, degree assortativity coefficient, modularity and degree centrality (see §1a), differs across various generative models. Ikehara *et al.* [12] and Canning *et al.* [14] have classified networks using network features like clustering coefficient (transitivity), mean degree and degree assortativity coefficient, which are subsets of our feature set. Our work advances these studies by incorporating a diverse array of theoretical network models, integrating spectral measures into the feature set and exploring a broader parameter range and combinations. Moreover, our study highlights the features’ impact and most importantly their interactions, which are less understood in the context of predictive modelling and classification tasks.

We have demonstrated the effectiveness of our pipeline, achieving nearly 100% prediction and classification accuracy alongside an impressive AUC score of nearly 1.0 across 10-fold cross-validation samples. Our classification accuracy outperforms the 94.2% of Canning *et al.* [14], 95% of Barnett *et al.* [13], Ikehara *et al.* [12], and between 55.7% and 91.4% that Nagy *et al.* had on their binary and multi-class classification model [24]. Our approach identifies distinct features distinguishing the prediction and/or classification of diverse networks, aligning closely with previous research [12,14,24]. However, the diversity of our approach makes it suitable for classification tasks as it enables a wide spectrum of structural properties observed in real-world networks such as sparsity, SW characteristics, clustering (transitivity), path length, degree distribution and subgraphs to be captured and estimated.

In addition, incorporating spatial networks enhances our approach [11]. The inclusion of spatial networks not only adds robustness to simulating large networks but also reproduces observed network properties such as sparsity, SW characteristics and clustering. Moreover, we provide compelling evidence that a sufficiently rich collection of structural features, particularly spectral measures like the (normalized) Fiedler value, eigenvector centrality and spectral radius, capture important structural heterogeneity in networks [22]. This allows our model to predict the generative model for network organization across different domains sharing similar structural properties. The exceptional performance of our pipeline negates the need for complex features like network motifs or graphlets employed in previous studies, which can incur considerable computational complexity [11,12,14,24].

Our findings across various generative models are consistent with well-established characteristics of theoretical networks [1,4,18,21–23,28]. For instance, ER networks, known for their randomness and low clustering [1,18,21–23,28], can also be accurately identified by emphasizing the often-overlooked spectral property, the normalized Fiedler.

Similarly, predictors such as degree centrality, eigen centrality and negative degree assortativity coefficient emerge as key indicators for SF networks, consistent with prior research [1,20,21,23,28]. In spatial networks, primary predictors include transitivity, mean eccentricity and diameter aligning well with prior studies [11,23,51]. Moreover, we have identified that spatial networks are associated with high eigenvector centrality, assortativity mixing and small normalized Fiedler due to its sparsity. Similarly, modularity, mean path length and positive degree assortativity coefficient are relevant in predicting the SBMs, aligning with findings from previous research [22,23]. Our study also identified spectral radius as a predictor of the SW network, an insight not initially known. Moreover, we found that the SW and SF networks are disassortative, while spatial and SBMs are mostly assortative.

Furthermore, we identified threshold values where interactions among network features influence predictions. Notably, key predictors like transitivity, spectral radius, modularity and normalized Fiedler remained important across a large range of training input over all simulations, consistently appearing among the top predictors for spatial, SW, SBMs and ER networks, respectively.

Our study highlights the importance of considering network features including spectral properties like the spectral radius, normalized Fiedler, as well as node relation network properties like the degree assortativity coefficient, alongside tailored predictors for each generative model. This approach facilitates effective network classification and enhances our understanding of the complex structure and dynamics of networks, indicating the robustness, community structure and potential pathways for processes like disease spread [6]. Overall, our analysis identified that features such as normalized Fiedler value, modularity, transitivity, degree centrality, spectral radius, degree assortativity coefficient and eigen centrality are the most relevant features for predicting the generative models.

While our study provides valuable insights, it is important to acknowledge certain limitations. Notably, we did not consider generative models such as 2K, forest fire, Kronecker graphs and exponential random graph models (ERGMs) [52–55]. Although these models may offer advantages in specific contexts like dynamic or evolving structures and nested hierarchical organizations found in certain networks [11,22,24,53,55], they may not be suitable for estimating empirical networks. For instance, Kronecker graphs suffer from scalability, model specification challenges and lack of realism and interpretability [53,54]. Similarly, ERGMs face scalability issues, model degeneracy and computational complexity when applied to large networks [55]. Moreover, ERGMs, 2K models and forest fire models all require significant computational time, and they produce complex parameter estimates that are difficult to interpret [55]. Conversely, the generative models we have chosen like ER, SBM, SF, SW and spatial, although some may seem simplistic like the ER networks, offer undeniable relevance in simulating real-world networks. While these models may not also capture certain properties as comprehensively as the excluded ones, they excel in reproducing fundamental network characteristics and offer better interpretability, computational efficiency and applicability to various network types, enabling meaningful insights into network organization and dynamics [1,19–21,28,56]. Ongoing future research aims to develop a hybrid generative model, such as a spatial SF-weighted model, capable of capturing a diverse range of properties from various networks. Additionally, potential future endeavours could delve into integrating the 2K, forest fire, Kronecker graphs and ERGMs [52–55] to deepen our comprehension of network formation and dynamics across diverse domains.

## 6. Conclusion

In conclusion, our method effectively identifies distinct features and their interactions in distinguishing the generative models. We achieved this by using novel interpretable machine learning tools like SHAP and Friedman and Popescu’s H-statistics [29–31]. Additionally, we utilized generative models as labels, enhancing our feature sets with spectral measures, simulating a substantial set of networks, and significantly expanding the parameter space over previous studies. Our results achieved an outstanding classification accuracy, nearly 100%, surpassing the performance of previous studies. Our pipeline, together with interpretable machine learning approaches, forms a potent tool for the identification of significant feature dependence and interactions and enhances our understanding of the underlying generative models. This in-depth understanding contributes to advancements in network classification and analysis, ultimately facilitating more accurate and effective modelling of complex systems. We hope that this work will provide both a useful tool and important insights into the analysis of biological and ecological networks.

## Data Availability

All the data and code to perform the analysis can be found at https://github.com/araimacarol/Empirical-Network-Classifier and have been archived within the Zenodo repository [57]. Supplementary material is available online [58].
